# 550 Retrospective Review of Fluid Resuscitation and Associated Complications for Adults in a Large Burn Center

**DOI:** 10.1093/jbcr/irad045.147

**Published:** 2023-05-15

**Authors:** Stacey Richerbach, Devin Nikjou, Suzanne Osborn, Van Dobbe, Natalie Kesler, Claudia Islas, Karen Richey, Kevin Foster

**Affiliations:** Arizona Burn Center at Valleywise Health, Phoenix, Arizona; AZ Burn Center at Valleywise Health, Phoenix, Arizona; AZ Burn Center at Valleywise Health, Phoenix, Arizona; AZ Burn Center at Valleywise Health, Phoenix, Arizona; AZ Burn Center at Valleywise Health, Phoenix, Arizona; AZ Burn Center at Valleywise Health, Phoenix, Arizona; Arizona Burn Center Valleywise Health, Phoenix, Arizona; AZ Burn Center at Valleywise Health, Phoenix, Arizona; Arizona Burn Center at Valleywise Health, Phoenix, Arizona; AZ Burn Center at Valleywise Health, Phoenix, Arizona; AZ Burn Center at Valleywise Health, Phoenix, Arizona; AZ Burn Center at Valleywise Health, Phoenix, Arizona; AZ Burn Center at Valleywise Health, Phoenix, Arizona; AZ Burn Center at Valleywise Health, Phoenix, Arizona; Arizona Burn Center Valleywise Health, Phoenix, Arizona; AZ Burn Center at Valleywise Health, Phoenix, Arizona; Arizona Burn Center at Valleywise Health, Phoenix, Arizona; AZ Burn Center at Valleywise Health, Phoenix, Arizona; AZ Burn Center at Valleywise Health, Phoenix, Arizona; AZ Burn Center at Valleywise Health, Phoenix, Arizona; AZ Burn Center at Valleywise Health, Phoenix, Arizona; AZ Burn Center at Valleywise Health, Phoenix, Arizona; Arizona Burn Center Valleywise Health, Phoenix, Arizona; AZ Burn Center at Valleywise Health, Phoenix, Arizona; Arizona Burn Center at Valleywise Health, Phoenix, Arizona; AZ Burn Center at Valleywise Health, Phoenix, Arizona; AZ Burn Center at Valleywise Health, Phoenix, Arizona; AZ Burn Center at Valleywise Health, Phoenix, Arizona; AZ Burn Center at Valleywise Health, Phoenix, Arizona; AZ Burn Center at Valleywise Health, Phoenix, Arizona; Arizona Burn Center Valleywise Health, Phoenix, Arizona; AZ Burn Center at Valleywise Health, Phoenix, Arizona; Arizona Burn Center at Valleywise Health, Phoenix, Arizona; AZ Burn Center at Valleywise Health, Phoenix, Arizona; AZ Burn Center at Valleywise Health, Phoenix, Arizona; AZ Burn Center at Valleywise Health, Phoenix, Arizona; AZ Burn Center at Valleywise Health, Phoenix, Arizona; AZ Burn Center at Valleywise Health, Phoenix, Arizona; Arizona Burn Center Valleywise Health, Phoenix, Arizona; AZ Burn Center at Valleywise Health, Phoenix, Arizona; Arizona Burn Center at Valleywise Health, Phoenix, Arizona; AZ Burn Center at Valleywise Health, Phoenix, Arizona; AZ Burn Center at Valleywise Health, Phoenix, Arizona; AZ Burn Center at Valleywise Health, Phoenix, Arizona; AZ Burn Center at Valleywise Health, Phoenix, Arizona; AZ Burn Center at Valleywise Health, Phoenix, Arizona; Arizona Burn Center Valleywise Health, Phoenix, Arizona; AZ Burn Center at Valleywise Health, Phoenix, Arizona; Arizona Burn Center at Valleywise Health, Phoenix, Arizona; AZ Burn Center at Valleywise Health, Phoenix, Arizona; AZ Burn Center at Valleywise Health, Phoenix, Arizona; AZ Burn Center at Valleywise Health, Phoenix, Arizona; AZ Burn Center at Valleywise Health, Phoenix, Arizona; AZ Burn Center at Valleywise Health, Phoenix, Arizona; Arizona Burn Center Valleywise Health, Phoenix, Arizona; AZ Burn Center at Valleywise Health, Phoenix, Arizona

## Abstract

**Introduction:**

The foundation for burn fluid resuscitation is well established, however an opportunity exists to improve outcomes for patients requiring such intervention. Resuscitation is influenced by a variety of factors, including associated complications. The purpose of this study was to establish a baseline at our center using historical performance and outcomes to drive quality improvement initiatives.

**Methods:**

This was a retrospective chart review of patients admitted over a five-year period requiring resuscitation. Due to factors, such as early enactment of comfort care, patients who expired < 48 hours of injury were excluded.

**Results:**

Charts were reviewed for 346 patients with a total body surface area (TBSA) >20%, 297 were adults. Of these, 32 died < 48-hours post-injury and 127 did not undergo fluid resuscitation or complete records were not available. Thus 138 were evaluable. Averages at admission were age 44.9, weight 86.4 kg and TBSA 39%. Most patients were male (71%). Mechanism was predominantly flame (86%), followed by 7% scald, 3% contact, 2% electrical, and 1% chemical. Concomitant inhalation injury was diagnosed for 34%. Average hourly fluid resuscitation volume was 6.5mL/kg/TBSA with a duration of 31.8 hours. Total volume exceeded Parkland formula calculations for 80%. Continuous albumin was administered for 97%, initiated on average at 8 hours post-injury. Boluses of lactated ringer’s and albumin were administered for 43% and 36% of patients, respectively. Urine output averaged 91 mL/hr and we identified a mean low optimal urine output of 43.2 mL/hr and mean high optimal output of 69.1 mL/hr.

Associated complications during fluid resuscitation included temperature < 36^◦^C (58%), vasopressor administration (12%), intra-abdominal pressure >12 (59%) and/or >19 (14%), and peripheral vascular pulses < +1 (70%). Escharotomy was performed for 42%; 32% preventative, 2% required subsequent fasciotomy. Laparotomy/open abdomen was performed for 7.9%. Diagnosed complications were low for compartment syndrome (9.5%), abdominal compartment syndrome (6.5%), and acute respiratory distress syndrome (1.4%). Continuous renal replacement therapy was initiated for 11.5% of patients < 48 hours post-burn and for 22.4% >48 hours; 25% requiring CRRT had a dialysis or renal history. The overall survival rate for this population was 76.1%. Patients were discharged most often to rehab (37%), home (19%), or skilled nursing facility (12%).

**Conclusions:**

A baseline for resuscitation in our center revealed a rate double historically accepted rates. Despite nonconformity with tradition, complications remain low. Outcomes have been favorable, yet opportunity for improvement endures.

**Applicability of Research to Practice:**

Further research is needed to evaluate the predictive value of assessment measures to forecast associated complications.

